# Falls in young adults: The effect of sex, physical activity, and prescription medications

**DOI:** 10.1371/journal.pone.0250360

**Published:** 2021-04-22

**Authors:** HyeYoung Cho, Michel J. H. Heijnen, Bruce A. Craig, Shirley Rietdyk

**Affiliations:** 1 Department of Health and Kinesiology, Purdue University, West Lafayette, IN, United States of America; 2 Center for Aging and the Life Course, Purdue University, West Lafayette, IN, United States of America; 3 School of Health and Applied Human Sciences, University of North Carolina Wilmington, Wilmington, NC, United States of America; 4 Department of Statistics, Purdue University, West Lafayette, IN, United States of America; São Paulo State University (UNESP), BRAZIL

## Abstract

Falls are a major public health issue not only for older adults but also young adults, with fall-related injuries occurring more frequently in adult females than males. However, the sex differences in the frequency and circumstances of falls in young adults are understudied. This research quantified the frequency and circumstances of falls as a function of sex, physical activity, and prescription medications in young adults. For 16 weeks, young adult participants (N = 325; 89 males; 19.9±1.1 years) responded to a daily email asking if they had slipped, tripped, or fallen in the past 24 hours. Falls and fall-related injuries were not uncommon in young adults: 48% fell at least once, 25% fell more than once, and 10% reported an injury. The most common activities at the time of the fall for females were walking (44%) and sports (33%), and for males, sports (49%) and walking (37%). A zero-inflated Poisson model revealed that higher number of falls were associated with the following: higher levels of physical activity (*p* = 0.025), higher numbers of medications (*p*<0.0001), and being male (*p* = 0.008). Regarding circumstances of falling, females were more likely to be talking to a friend at the time of the fall (OR (95% CI): 0.35 (0.14–0.73); *p* = 0.01). For slips and trips without a fall, males and females reported the same number of slips (OR (95% CI): 0.885 (0.638–1.227) p = 0.46), but females reported more trips (OR (95% CI): 0.45 (0.30–0.67); *p*<0.01). Only females reported serious injuries such as concussion and fracture. In conclusion, the rate of falls in young adults was affected by physical activity levels, number of medications, and sex. Quantifying and understanding these differences leads to increased knowledge of falls across the lifespan and is instrumental in developing interventions to prevent falls.

## Introduction

Recent research has demonstrated that falls are a serious concern not only for older adults, but also for young adults [1–6]. The total cost of fall-related injuries for adults aged 18–24 in the US exceeded $7 billion in 2010 [6]. The majority of research in young adults has examined fall-related injuries obtained from medical reports [1, 3] and injury self-report [2]. However, these approaches do not capture all falls, only those with injuries. A more complete understanding of falls in young adults can be obtained by quantifying all falls, including non-injurious and injurious falls.

**Fig 1:**
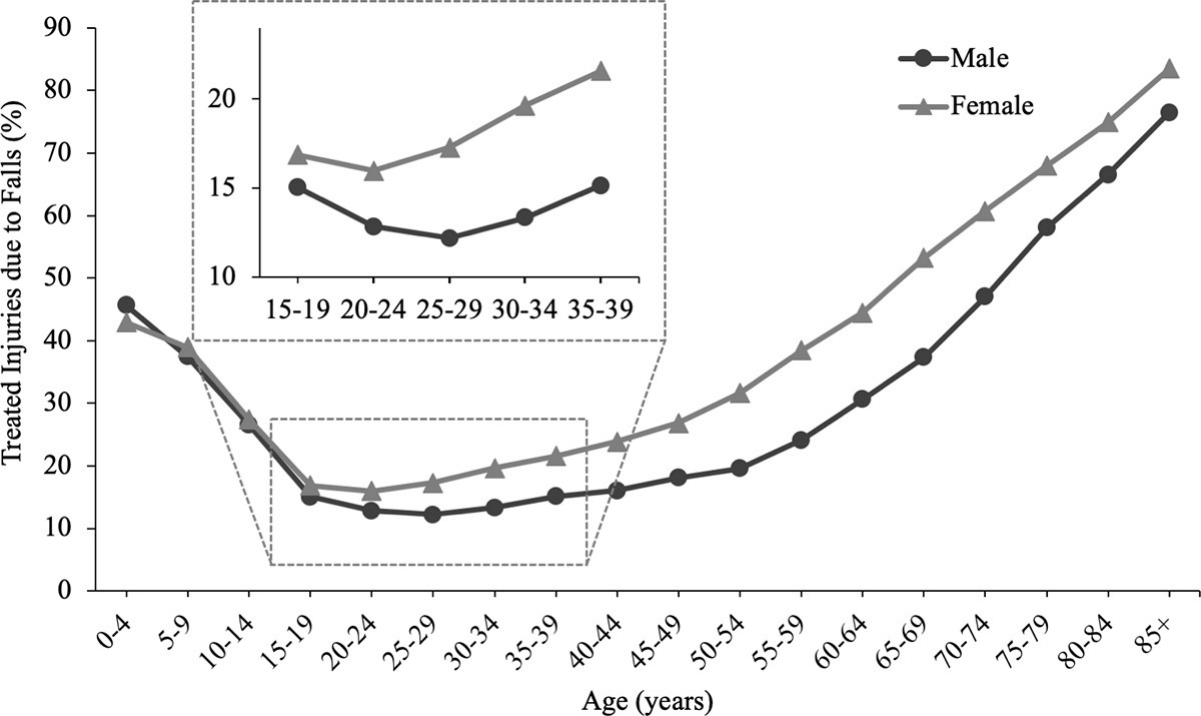
Percent of all medically treated injuries that resulted from falls as a function of age and sex for 2015. Data extracted from the CDC’s Web-based Injury Statistics Query and Reporting System (WISQARS^TM^) for non-fatal injuries [7]. Inset shows greater detail for ages 15–39 years.

While it is well-known that older females sustain more injuries than older males (Fig 1; [7, 8]), the higher injury rate for females is first evident at age 20 (Fig 1 inset). In young adults aged 20–45 years, females were more likely to report a fall than males (20 vs 17%, respectively), and were more likely to be injured from the fall (81 vs 61%, respectively) [9]. Extending these analyses to a more restricted age range will provide insights at the age when differences in fall injuries are first noted between females and males (20 years, Fig 1 inset). Examining sex-related differences in falls in young adults is especially important as young females (aged 20–29 years) had a 25% increase in fall-related fractures in the 10 year period from 2000 to 2010, while in males of the same age, the increase was 5% (not statistically significant) [1]. Furthermore, females had a higher fall-related injury rate on stairs at all ages except ≤ ten years [3], and the proportion of fall-related injuries on stairs was highest among young females [2].

Young adults are more active than older adults [10], and a higher activity level in young adults was associated with a higher frequency of falls [4]. This likely results from higher sports participation in young adults [11], which increases exposure to hazardous situations [12]; this increased risk is supported by the observation that sports-related falls are common in young adult males [2, 9]. Conversely, for older adults, higher levels of physical activity are generally considered as protective against falls (e.g. [13]). Quantifying the association between physical activity and falls in young adults will increase our understanding of the factors related to falls across the adult lifespan.

Another factor that may be relevant to falls in young adults is number of prescription medications. Prescription medications have been linked to falls in older adults (e.g. [14, 15]), and to fall-related injuries in adults aged 25–60 years [16]. While the latter study includes young adults, the age range spans four decades. It is important to examine a more restricted age range, to determine if the association between number of medications and falls is also evident when only young adults are included in the sample.

Overall, while previous research has demonstrated that sex, physical activity, and number of medications are associated with falls and fall-related injuries in young adults [2–6, 9, 16], no studies have examined these factors concurrently. The primary purpose of this study was to determine if the frequency of falls was associated with sex, physical activity level, and number of medications in college-aged young adults. Fall frequency was quantified with a daily on-line survey [4] as contemporaneous fall reports given at regular intervals (i.e. prospective) are more accurate than retrospective reports [17–20]. The daily survey also allowed us to quantify fall circumstances (e.g. activity at time of falls, injury), and slips and trips that did not result in falls. While slips and trips are common precipitating causes of falls and fall-related injuries across the adult lifespan [2, 4, 19, 21–23], the frequency of slips and trips during daily activities is understudied. These observations in young adults will increase knowledge that may lead to new intervention targets to mitigate the high costs associated with falls.

## Methods

**Fig 2:**
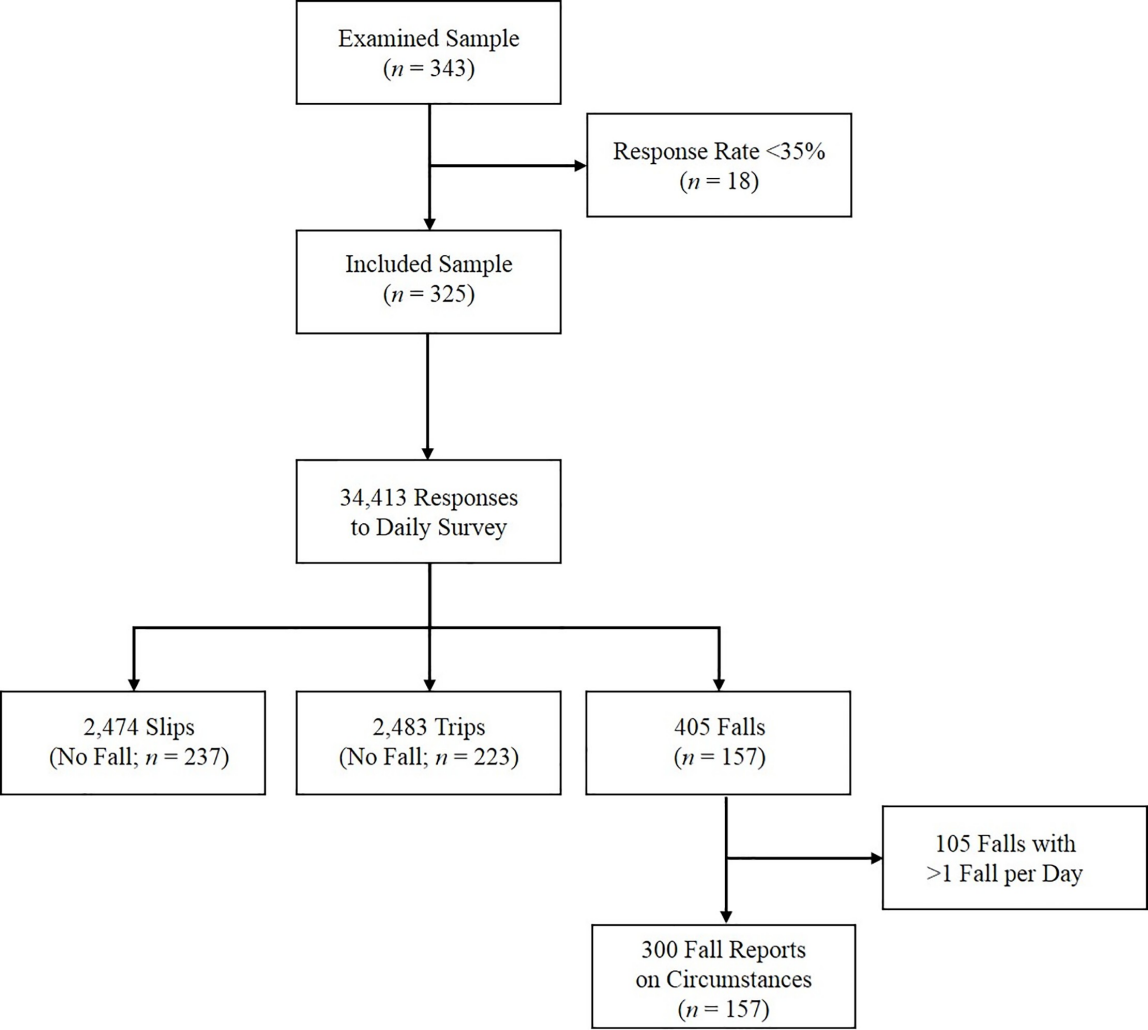
Participant flow diagram. Figure also includes the number of responses to the daily survey, number of slips and trips reported without a fall, number of falls, and number of reports on fall circumstances.

**Fig 3:**
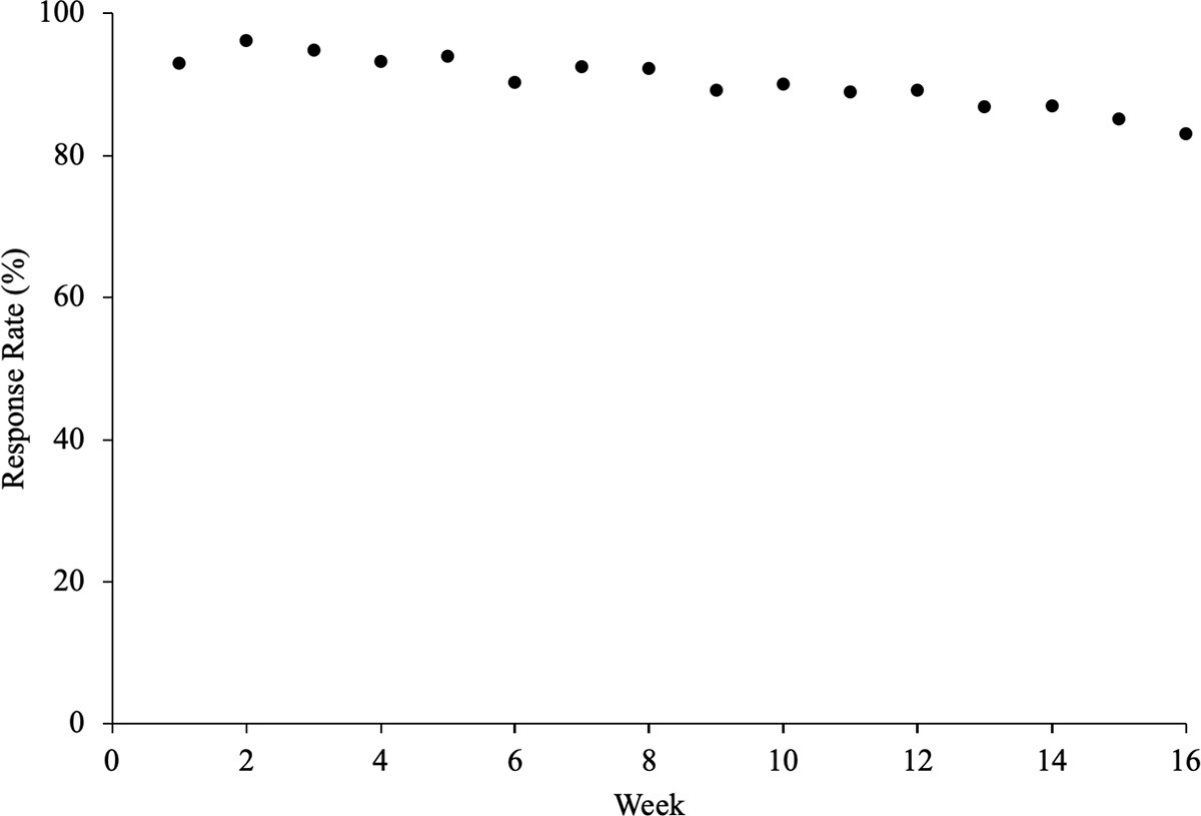
Survey response rate as a function of week. Eighteen participants who had a response rate less than 35% are included.

Undergraduate students (N = 343; 96 males) participated in this study for one academic semester (16 weeks). The undergraduate students enrolled in the department (Health and Kinesiology) are 64% female, consistent with the higher percentage of female participants (72%) observed in the study. The results from the first 94 subjects were previously published [4]. All students were taking the same course, and were offered 1% extra credit for (A) participating in the survey or (B) completing a short written assignment. The goal of the alternate assignment was to limit potential coercion to participate in the research. Of the 510 students enrolled in the course over nine semesters (from 2014–2019), 343 chose to participate in the survey (67%) (Fig 2). No instruction regarding required participation in the daily surveys was provided; all students who signed the online consent received 1% extra credit whether or not they responded to the daily emails. The response rate of the 343 participants ranged from 0 to 100%, with mean, median, and mode of 91%, 98%, and 100%, respectively (response rate was calculated for each participant as (number of responses/number of daily surveys*100)). Weekly response rate is shown (Fig 3). Eighteen participants (7 males) were excluded from further analyses because they responded to less than 35% of the daily emails [4] (Fig 2); six of these 18 participants did not respond to any daily surveys after signing the online consent form. In the remaining 325 participants, the response rate ranged from 38–100%. These 325 participants (age 19.9±1.1 years, range 18–27 years, 89 males; height 169.9±9.3 cm; weight 68.9±15.1 kg) were included in the following results. The informed consent and study methods were approved by the Human Research Protection Program.

Two types of surveys were distributed via Qualtrics Survey Software (Qualtrics Labs Inc. Provo, UT): (1) initial survey distributed once for consent and demographic information and (2) daily survey distributed daily for 16 weeks. These surveys are available as supplemental content in [4]:

### Initial survey (distributed once in week 1)

The initial survey included demographics (e.g. age, sex, height, weight, number of prescription medications, physical activity level), and was distributed by email once in the first week. For the number of prescription medications, participants were instructed to select one of the follow six categories: 0, 1, 2, 3, 4, 5 or more. Two subjects (females) selected 5 or more. Since we could not determine the exact number of medications, these two subjects were removed from analyses involving medication. The number of prescription medications, rather than drug name or category, was selected for several reasons. First, many patients cannot name any of their medications [24], and if students completed the on-line survey away from home, medication information may not have been available. Second, the number of prescription medications were associated with increased risk of fall-injury in young and middle-aged adults [16]. Third, the number of prescription medications increased fall-risk even after adjusting for fall-risk-inducing drugs in older adults [15]. Therefore, in this study, the number of prescription medications were quantified, not medication name or category. Physical activity was assessed with the Leisure-Time Exercise Questionnaire (LTEQ) [25]. The LTEQ is a short survey that quantifies frequency of participation in three categories of physical activity (strenuous, moderate, and mild) with at least 15 minutes duration for an average week. The LTEQ provides a single score which ranks physical activity levels in individuals [26].

### Daily survey (distributed daily for 16 weeks)

Each morning, participants received an emailed survey that asked “Have you slipped, tripped or fallen in the past 24 hours?”. The following definitions were provided with the question: “FALL–an undesired contact of any body part (other than the feet) with the ground or a lower surface” [27]; “TRIP–your foot or shin contacted the ground or other object unexpectedly”; and “SLIP–your foot slipped while in contact with the ground”. If the participant responded ‘No’, the survey concluded. If ‘Yes’ was selected, the participant was asked to enter the number of occurrences of each trip, slip, or fall in the past 24 hours. When a fall was reported, questions regarding the circumstances were also presented. Circumstances included activity, precipitating cause, time of day, injuries and other factors. If the participant reported more than one fall in the previous 24 hours, participants were asked to describe the circumstances of the fall that were perceived as the most serious or interrupted their movement the most. Limiting the survey to one fall a day was done to mitigate survey fatigue observed in pilot research.

### Data analyses

The frequency of falls was quantified with two approaches: (1) with all falls included and (2) with sports-related falls excluded. The latter categorization (sports-related-falls-exclusion analysis) was completed because sports are associated with a higher risk of falls, and avoiding falls during sports may be impossible. For the sports-related-falls-exclusion categorization, when more than one fall occurred in a day, it was assumed that all falls on that day occurred for the same reason as the one fall that had an associated fall report. Thus, if two falls occurred on one day, and the reported fall was due to sports, both falls were eliminated in the sports-related-falls-exclusion categorization.

### Statistical analyses

#### Fall frequency

We used a zero-inflated Poisson model to describe the relationship between number of falls and sex, activity level (LTEQ score), and number of prescription medications. We selected a zero-inflated Poisson model because of a larger than expected number of participants (under the Poisson distribution) with zero falls. The model included two and three-factor interactions (sex, physical activity, and number of medications) and we used AIC to select the best model. The LTEQ and number of medications were centered (the mean of all values was removed from each individual value). Centering was completed in order to reduce the collinearity between the interaction term and the associated main effect terms. The number of response days for each participant (up to 112 responses: 7 surveys a week for 16 weeks) was used as an offset variable to account for differences in sampling intensity.

#### Fall circumstances

Three hundred fall reports were obtained from 157 participants (Fig 2), indicating that some participants contributed more than one fall report. Fall circumstances (e.g. activity at time of fall, cause of fall, injury) were quantified for all participants and also separately for males and females. A bias-corrected and accelerated (BCa) bootstrap interval of the odds ratio (OR; males divided by females) was constructed (bivariate analysis). The BCa analysis accounted for the fact that each participant could contribute multiple falls (Fig 2). Confidence intervals were not reported for cases when the count for either sex was less than 5, or when the category was “other.”

#### Slip and trips without falls

The probability of reporting a slip or a trip *without an associated fall* was examined with a mixed logistic regression, and the OR was determined for males versus females (males divided by females).

The zero-inflated Poisson model and mixed logistic regression were completed in SAS 9.3 (Cary, NC, USA), and the BCa bootstrap interval of the OR was completed in R (RStudio, Boston, MA, USA). Significance level was set at *p* ≤ 0.05.

## Results

### Response rate of 343 participants

The response rate decreased from 95% (first two weeks) to 84% in the last two weeks, with an average of 90% (Fig 3). Male response rate was not different from females (89 and 91%, respectively; OR (95% CI): 1.25 (0.70–2.25); p = 0.45). The following results do not include the 18 subjects with response rates lower than 35%, as described in the methods. From the 325 subjects, a total of 34,413 responses to Question 1 of the daily survey were obtained (Did you slip, trip or fall in the past 24 hours?) (Fig 2).

### Fall frequency: Descriptive summary

**Fig 4:**
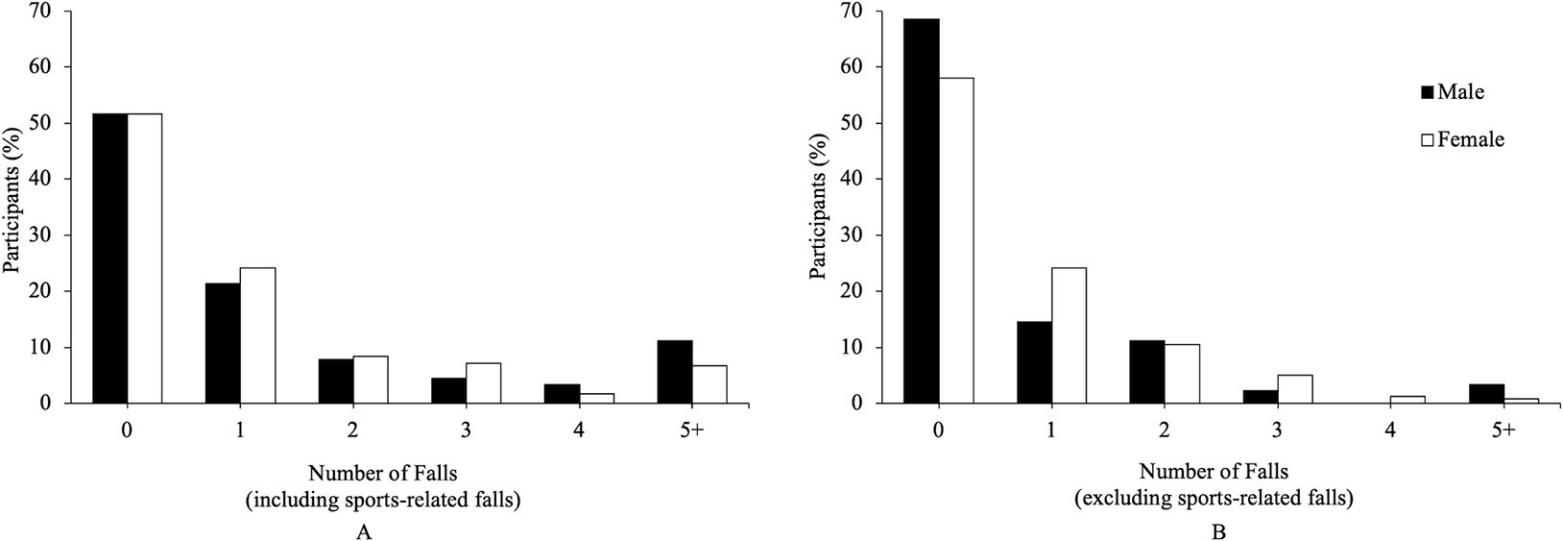
Percentage of participants versus the number of reported falls with sports-related falls included (A) and sports-related falls excluded (B). Eighteen participants with a response rate less than 35% are not included.

Four hundred and five falls were reported by 157 subjects (48% of 325 subjects) (Fig 2), with 115 falls reported by 43 males (48%) and 290 falls reported by 114 females (48%). A completed fall-circumstance report was available for 300 of the 405 falls (74% of all falls); a fall-circumstance report was not completed for 105 falls (26%) due to participants falling more than once in a day (see methods). There were 34 reports of two falls in one day, and 21 reports of more than two falls in one day. Eighty-one subjects (25%) fell more than once in the 16-week interval (termed frequent fallers). When examined as a function of sex, 43 males (48%) and 114 females (48%) fell at least once, and 24 males (27%) and 57 females (24%) fell more than once (Fig 4A).

### Fall frequency: Effect of sex, physical activity, and number of medications

**Fig 5:**
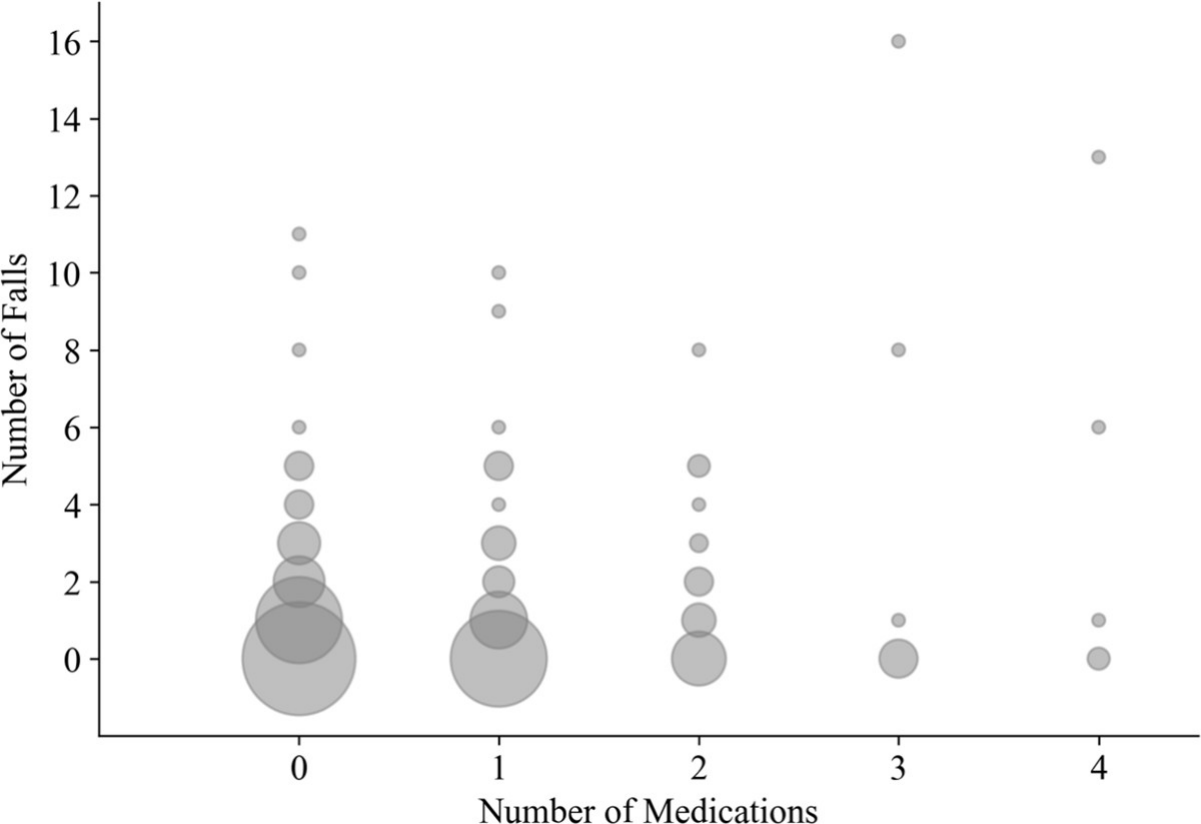
Bubble plot of the number of medications versus the number of falls. With a zero-inflated Poisson model, number of medications was significant in both Poisson (*p*<0.0001) and zero-inflation distributions (*p* = 0.025) (see also Table 1). While the mean number of falls increased with number of medications, this effect was dampened by a greater chance of zero falls: mean number of falls increased 23% for 0 to 1 medications, 21% (1 to 2 medications), 19% (2 to 3 medications), and 16% (3 to 4 medications).

For *frequency of falls*, we started with a zero-inflated Poisson model that included two and three-factor interactions and used AIC to select the best model. This resulted in all three variables in the Poisson component: sex (*p* = 0.008), physical activity (LTEQ) (*p* = 0.025), and number of medications (*p*<0.0001), and only the number of medications in the zero-inflation component (*p* = 0.025) (See Table 1 for parameter estimates and CI). Holding the other two factors fixed, a male’s average number of falls was 38% higher (95% CI: 8.0–89.0%) relative to a female, and an increase of 10 points on LTEQ increased the mean number of falls by 4% (95% CI: 0.7–8.0%) (Ten LTEQ points would be approximately equivalent to 15 minutes each week of strenuous exercise, 30 minutes of moderate exercise, or 45 minutes of light exercise). As noted above, number of medications was significant in both Poisson (*p*<0.0001) and zero-inflated distributions (*p* = 0.025) (Fig 5), indicating that while the mean number of falls increased with number of medications, this effect was dampened by a greater chance of zero falls. Thus, the mean number of falls increased 23% for 0 to 1 medications, 21% (1 to 2 medications), 19% (2 to 3 medications), and 16% (3 to 4 medications).

**Table 1:** Parameter estimates and 95% CI for zero-inflated and Poisson components of the zero-inflated Poisson model.

	Zero-Inflation Component			Poisson Component		
	Estimate	95% CI	*p*	Estimate	95% CI	*p*
Intercept	-0.3066	-0.6564–0.0432	0.0858	-3.9832	-4.1351– -3.8313	**<0.0001**
Medications	0.3214	0.0413–0.6015	**0.0245**	0.3367	0.2349–0.4384	**<0.0001**
Sex				0.3529	0.0926–0.6132	**0.0079**
Physical Activity				0.0041	0.0005–0.0077	**0.0250**

### Falls that were not associated with sports: Descriptive summary

Since fall-risk is higher during sports activities, fall frequency was also examined without sports. Two hundred and twenty-two non-sports-related falls were reported by 127 subjects (39% of 325 subjects), with 57 subjects (18%) reporting more than one fall (Fig 4B). When examined as a function of sex, 28 males (31%) and 99 females (42%) fell at least once, and 15 males (17%) and 42 females (18%) fell more than once (Fig 4B).

To determine if the frequent fallers were more likely to fall during sports, the activity at the time of the fall was examined as follows. Sixty-seven of the 81 frequent fallers (83%) reported at least one fall that was not related to sports. Thirty-nine of the 81 frequent fallers (48%) had no falls related to sports. Fifteen of the 81 frequent fallers (19%) reported 100% of their falls as due to sports.

### Causes of falls

The four main causes of falls were slip (38%), trip (29%), hit/bump (11%), and loss of support with object (7%) (Table 2). When an environmental cause was selected, ice/slippery surface (34%) and stairs (13%) were the main environmental causes for all falls. When only non-sports related falls were considered, ice/slippery surface (44%) and stairs (22%) were the main environmental causes. The majority of ice/slippery surface falls occurred outdoors (88%), while the majority of stair-related falls occurred indoors (69%). Stair-related falls occurred at a similar percentage for ascent (52%) and descent (48%).

**Table 2:** Fall circumstances for all fallers, and comparison for male versus female fallers.

	Number of Falls (percent)			OR	
	Total Fallers (N = 157, 300 falls)	Male Fallers (N = 43, 73 falls)	Female Fallers (N = 114, 227 falls)		95% CI
**Cause of falls:**					
Slip	113 (38)	29 (40)	84 (37)	1.122	0.576–2.266
Trip/stumble	87 (29)	23 (32)	64 (28)	1.172	0.503–2.406
Hit/Bump	33 (11)	11 (15)	22 (10)	1.653	0.453–4.743
Loss of Support with object	20 (7)	5 (7)	15 (7)	1.039	
Other	47 (16)	5 (7)	42 (19)		
**Injuries:**					
Injured	43 (14)	7 (10)	36 (16)	0.563	0.396–1.318
Contusion	20 (7)	2 (3)	18 (8)	0.327	
Abrasion	17 (6)	4 (5)	13 (6)	0.954	
Strain/sprain	8 (3)	2 (3)	6 (3)	1.038	
Concussion	2 (1)	0 (0)	2 (1)		
Fracture	1 (0)	0 (0)	1 (0)		
Other injuries (e.g. soreness)	6 (2)	2 (3)	4 (2)		
Medical Treatment	3 (1)	0 (0)	3 (1)		
**Activity at time of fall:**					
Walking	126 (42)	27 (37)	99 (44)	0.739	0.394–1.464
Sport	111 (37)	36 (49)	75 (33)	1.947	0.991–3.890
Running/jogging	30 (10)	6 (8)	24 (11)	0.757	0.251–1.770
Transfer	10 (3)	2 (3)	8 (4)	0.771	
Other	23 (8)	2 (3)	21 (9)		
All activities except sport	189 (63)	37 (51)	152 (67)	0.507	0.264–1.027
**Concurrent task:**					
No Concurrent Task	194 (65)	57 (78)	137 (60)	2.009	0.896–4.559
Talking to friend	74 (25)	9 (12)	65 (29)	**0.350**	**0.139–0.728**
Texting	6 (2)	1 (1)	5 (2)	0.616	
Talking on phone	2 (1)	2 (3)	0 (0)		
Other	24 (8)	4 (5)	20 (9)		
**Environment:**					
Ice/slippery	102 (34)	25 (34)	77 (34)	1.015	0.568–1.969
Nothing in environment	64 (21)	16 (22)	48 (21)	1.047	0.454–2.205
Stairs (up and down)	40 (13)	7 (10)	33 (15)	0.582	0.166–1.461
Footwear	16 (5)	1 (1)	15 (7)	0.196	
Other	90 (30)	29 (40)	61 (27)		
**Alcohol/Drugs**	33 (11)	8 (11)	25 (11)	0.994	0.345–2.364
**Lighting**					
Well lit	197 (66)	47 (64)	150 (66)	0.928	0.477–1.920
Poorly lit	97 (32)	25 (34)	72 (32)	1.121	0.531–2.234
**Outdoor vs. Indoor**					
Outdoor	172 (57)	43 (59)	129 (57)	1.089	0.539–2.231
Indoor	119 (40)	28 (38)	91 (40)	0.930	0.423–1.875
**Time of day**					
6:00 AM–12:00 PM	57 (19)	8 (11)	49 (22)	0.608	0.238–1.294
12:00 PM–6:00 PM	86 (29)	23 (32)	63 (28)	1.197	0.594–2.401
6:00 PM–12:00 AM	123 (41)	32 (44)	91 (40)	1.166	0.633–2.118
12:00 AM–6:00 AM	28 (9)	8 (11)	20 (9)	1.274	0.430–3.301

Odds ratio (OR) and 95% confidence interval (CI) for males versus females (bivariate analysis). OR not calculated when either observation was zero, or when the category was other. CI not calculated when either observation was five or less, or when the category was ‘other’. Significant difference bolded (*p*<0.05).

### Activity at time of fall

The top three activities for males were: sports, walking, running/jogging; for females: walking, sports, running/jogging (Table 2). Thirty-five percent of falls occurred while multitasking. The most common multitask was talking to a friend, which occurred in 25% of the falls. Few falls occurred while texting (2%) or talking on phone (1%) (Table 2). Of the six falls during texting (five females, one male), three occurred on stairs and two on icy/slippery surfaces.

### Injuries from falls

Forty-three falls in 34 participants resulted in an injury (14% of falls, 10% of participants) (Table 2). Three falls resulted in a serious injury (broken bones or concussion) (1% of falls).

### Falls related to alcohol and drugs

Alcohol or drugs were reported to be related to the fall in 33 falls (11% of falls).

### Other circumstances associated with falls

The majority of falls occurred outdoors (57%) and in bright light circumstances (66%). The most common (41%) time when falls occurred was between 6 pm and 12 am.

### Fall circumstances: Effect of sex

As noted in the methods, the effect of sex on fall circumstances was examined statistically only where there were sufficient observations to conduct the analyses. One circumstance was significantly different across sex: Females were significantly more likely to report talking to a friend (OR = 0.350, Table 2). While the remaining circumstances were not significantly different (or did not have sufficient observations for comparison), there were several trends that are noteworthy and should be considered for follow-up research with larger numbers of participants. Males demonstrated a trend where they were more likely to fall during sports activities than females (49% vs 33%; Table 2), and, similarly, females demonstrated a trend where they were more likely to fall during activities outside of sports (67% vs 51%; Table 2). Females demonstrated a trend of being more likely to fall on stairs (15% vs 10%; Table 2). Females had a trend of being more likely to report the fall as related to footwear (7% vs 1%; Table 2).

Regarding injuries, a trend was observed where females were more likely to report an associated injury than males (16% vs 10% of falls; Table 2). A fall-related injury was reported in 7% of all male participants and 12% of all female participants. Six males reported 10 injuries in 7 falls (10% of falls); 28 females reported 44 injuries in 36 falls (16% of falls) (Table 2). Males reported two contusions (29% of injuries), four abrasions (57%), and two sprain/strain injuries (29%). Females reported 18 contusions (50%), 13 abrasions (36%), six sprain/strain (16%), two concussions (6%), and one fracture (3%). In males, fall-related injuries occurred during sports (86% of injuries) and running (14% of injuries). In females, fall-related injuries occurred during walking (39% of injuries), sports (36%), running (19%), and standing/transfer (6%). Medical treatment was obtained for three falls (7% of all injuries, 1% of all falls); only females reported medical treatment.

### Slips and trips that did not result in a fall

During the 16 weeks, 2474 slips and 2483 trips that did not result in a fall were reported (average: 1.0 reported perturbations per week per participant, with 0.5 slips and 0.5 trips per week per participant). Two-hundred and thirty-seven participants (57 males, 180 females; 73% of all participants) reported at least one slip and 223 participants (50 males, 173 females; 60% of all participants) reported at least one trip, where neither perturbation resulted in a fall. The reported slips were not different for males versus females (*p* = 0.46, Table 3), but females were more likely to report a trip (*p*<0.01, Table 3) (average: 0.3 and 0.6 reported trips per week per male and female participant, respectively). Of all reported slips (with and without falls), 6% resulted in a fall, and of all reported trips, 4% resulted in a fall.

**Table 3:** Frequency of reported slips and trips–that did not result in a fall–for males versus females.

	Number of Perturbations Without Falls (Percent of all Reports)				
	All	Male	Female	OR	95% CI
	(N = 325; 34,113 observations)	(N = 89; 9,395 observations)	(N = 236; 24,718 observations)		
Slips	2474 (7)	719 (8)	1755 (7)	0.885	0.638–1.227
Trips	2483 (7)	370 (4)	2113 (9)	**0.446**	**0.297–0.670**

Odds ratio (OR) and 95% confidence interval (CI) for males versus females. Significant differences bolded.

## Discussion

The primary purpose of this study was to determine if falls are associated with sex, physical activity level, and number of medications in young adults. Current knowledge was extended with a protocol that: (1) focused on a discrete age group, 18–27 years, and (2) included daily contemporaneous reports (i.e. prospective fall reports). Forty-eight percent of participants reported a fall, 14% of falls resulted in an injury, and 1% of falls required medical treatment. We observed that the frequency of falls was positively associated with physical activity level, number of medications, and being male. Females were more likely to be talking to a friend when a fall occurred and females were more likely to report a trip without a fall. Although there were not enough observations to run statistical analyses, only females sustained serious injuries (fractures and concussions) that required medical treatment.

Consistent with our previous findings [4] and recent research on fall-related injuries [1–3, 5, 6], falls in young adults were not uncommon and resulted in injuries. While about a third of these falls occurred due to participation in sports activities (where fall risk is higher), young adults also fell outside of sports as the following two observations emphasize. First, when sports-related falls were excluded, percent fallers remained relatively high at 39%. Second, while it seems reasonable to expect that the frequent fallers (25% of participants) were falling during sports, 83% of the frequent fallers fell at least once outside of sports (Fig 4B). Fall-related injuries were not uncommon in young adults: 10% of all participants reported an injury, 14% of falls resulted in an injury, while 1% of all participants and 1% of all falls resulted in injuries that were medically treated (one fracture and two concussions) (Table 2). Compared to a previous study in young adults (20–45 years, *n* = 292) [9], the percent fallers was more than twice as high (48 vs 18% of participants), and the percentage of falls with injuries was about five times lower (14% vs 72% of falls). The differences are likely due to the approach (prospective daily online survey vs retrospective report from past two years; retrospective reports are less accurate [17–20]), and the narrower range of ages observed here (18–27 vs 20–45 years). Overall, the results support the growing contention that falls are a serious concern for young adults [1–6].

The most common precipitating causes of falls were slips and trips (Table 2), which were also the most common causes of fall-related injuries in young adults [2] and are common causes of falls and fall-related injuries in older adults [19, 21–23]. Slips and trips were common in the young adults observed here, with an average of 0.5 slips and 0.5 trips per week in young adults (Table 3). However, young adults had sufficient balance skills to recover from the vast majority of these perturbations (94% of slips and 96% of trips did not result in a fall). These values are similar to slips in restaurant workers, where 0.4 slips were observed in 40 work hours, and workers recovered from 94% of slips [28]. The observation that trips occurred commonly in the field is consistent with observations in lab-based locomotor experiments: When stepping over stationary, visible obstacles in the lab, participants inadvertently contact the obstacle about 1–2% of the times they step over the obstacle (see summary in [29]). Overall, the observations reported here support the growing body of research that slips and trips are commonly experienced perturbations [4, 28, 29], and they are common causes of falls and injuries across the adult lifespan [2, 4, 19, 21–23]. These findings support the continued development of perturbation training where people are trained to recover from falls by safely exposing them to slips and trips (e.g. [30]). Furthermore, the training may have benefit not only for balance-compromised populations, but also young healthy adults, since they regularly experience slips and trips and these perturbations are the most common cause of falls.

Higher levels of physical activity in young adults were associated with higher frequency of falling (Table 1), likely indicating increased exposure to hazardous situations [12]. Conversely, in older adults, higher levels of physical activity are generally associated with decreased falls, reflecting the positive effect of physical activity on age-related changes in balance (e.g. [13, 31, 32]). However, even in older adults, engaging in sports or vigorous-intensity physical activity was associated with increased falls [12, 31, 33]. Falls related to vigorous-intensity activity may be perceived as less important since these falls are hard to avoid and can be considered self-imposed. However, vigorous physical activities maximize health benefits (e.g. [34]), and national guidelines recommend moderate-to-vigorous activity in all ages [13]. Since vigorous activities are recommended for population health, it is important to understand the association between sports/vigorous-intensity activity and falls/fall-related injuries observed here and in earlier research [2, 9, 12, 31, 33]. Follow-up studies are recommended to (1) identify which sports and activities are more likely to lead to injuries, (2) determine possible steps for remediation within a sport or activity, and (3) identify individual factors that increase likelihood of injury during sports and vigorous activities.

A positive association was observed between number of falls and number of medications in this young adult sample (Fig 5), which is consistent with associations commonly observed in older adults (e.g. [15, 35]). This association has also been observed in a sample of young and mid-life adults (25–60 years) [16]; we demonstrate here that the association was also evident in a younger, narrower age range (18–27 years). While our study protocol does not allow us to determine if the association is due to underlying comorbidities or to the medications, it is evident that using one or more medications resulted in higher risk of falling. This is a largely unrecognized relationship in young adults that should be explored further and considered in fall prevention programs.

One of the goals of this study was to understand why young adult females sustain more fall-related injuries (Fig 1) [2, 3, 9]. While the number of injuries was not significantly higher in the current data, the trend indicated more injuries were observed in females (16% of females and 10% of males reported a fall-related injury, respectively; Table 2), consistent with previous research [2, 3, 9]. Higher injuries in females could result from the following possibilities: (1) females are more likely to be injured from a fall (where males and females fall at similar rates), and/or (2) females and males are equally likely to be injured from a fall, but females fall more frequently. The current observations do not support the idea that females sustain more injuries because they fall more frequently. Rather, we observed similar percentages of male and female participants that reported at least one fall (48% and 48%, respectively) and males reported a higher number of falls than females (Table 1). While males reported higher number of falls, we argue it is unlikely that females have better balance-ability relative to males based on several observations. These observations include: females reported twice as many trips without an associated fall, and females were more likely to be talking to a friend at the time of a fall than males. Furthermore, the following trends also do no support the idea that females have better balance than males: females were more likely to fall during lower risk activities, and females reported more injuries during walking (males reported no walking injuries). These observations are described more fully below.

The higher number of trips reported by females (Table 3) may indicate impaired gait control relative to males. Tripping results from inappropriate foot placement around an obstacle and/or insufficient foot elevation to clear the ground or an obstacle [29, 36, 37]. However, it is important to note that since the number of trips were obtained from self-report, an alternative explanation is that females were more likely to *perceive* trips than males, rather than actually experiencing more trips. Perception may be affected by sex through factors such as higher levels of overconfidence in males [38]. We believe that the perception explanation is unlikely, because both sexes reported similar numbers of slips (Table 3), and it is unlikely that males would be more likely to perceive slips than trips. Therefore, it seems more likely that females tripped more, rather than females perceived more trips. Furthermore, a parallel is noted between the higher trip rate found here in young females and the observation that, across the adult lifespan, females were more likely than males to sustain an injury from a trip [2]. These parallel observations lead to the speculation that increased likelihood of tripping is a cross-age phenomenon for adult females (although the younger counterparts are less likely to sustain injury (Fig 1)).

Females were twice as likely as males to report talking at the time of the fall (Table 2), indicating that females may be more impaired during locomotor multitasking. Walking while talking is cognitively demanding not only for older adults [39], but also for young adults [40], due to the concurrent management of ongoing locomotion, terrain navigation, language formulation, and speech generation. This cognitive demand may be greater in females due to a differential focus on social versus physical cues [41, 42]. Road-crossing observations indicate that males focused more on physical cues (such as vehicles), while females focused more on social cues (such as other pedestrians), based on self-report measures [41] and gaze behavior [42]. If road crossing observations can be extrapolated to everyday tasks, males may attend more to the physical environment and be more likely to note upcoming hazards such as curbs and stairs, while females may attend more to social cues, such as the person they are talking to, and not sufficiently attend to the physical environment. Note that sex-related differences in gaze behavior may also explain why females are more likely to trip (Table 3), as females may not pay sufficient attention to the location and size of an obstacle. However, we note that higher reports of talking at the time of the fall by females has at least three possible explanations: 1) females were more balance-compromised than males when they were talking, as described above, 2) females were more likely to be talking than males during daily activities, and they happened to be talking when they fell, and/or 3) females were more likely to remember talking than males. Our protocol does not allow us to determine which possibility is accurate; lab-based research examining sex-related differences during locomotor multitasks is required.

The trends of (1) females falling during lower risk activities and (2) females reporting more injuries during walking (Table 2) are also consistent with impaired balance in females. The most common activity at the time of the fall for males was sports (49%), where falls are more likely to occur and perhaps even expected. Conversely, the most common activity for females was walking (44%), a lower-risk activity where falls are not expected in healthy adults. Although this trend was not statistically significant, it is consistent with previous research: The most common activity at the time of the fall was also sports-related for males and walking/ambulation for females (with fall self-report for ages 20–45 years [9] and with fall-injury medical reports for ages 18–44 years [2]). Therefore, consistent sex-related observations regarding activity at time of the fall for young adults were obtained in three different studies with different protocols, emphasizing that females are more likely to fall during lower risk activities, while males are more likely to fall during higher risk activities. Regarding activity during fall-related injuries, males reported no injuries during walking, while females reported that 38% of injuries occurred during walking (Table 2). All of the injuries in males occurred during the higher-risk activities of sports (86%) or while running/jogging (14%). Thus, there are a series of observations that support the idea that females have impaired balance/gait control relative to males, although the results do not definitely identify why females sustain more injuries than males (Fig 1).

The sex-related differences leading to impaired balance/gait control in young adult females could result from the following: lower muscle mass and strength [43], slower and more variable reaction time [44], lower bone mass and skeletal integrity [45], greater focus on social cues (versus physical cues) [41, 42], and higher likelihood of wearing ‘poor shoe types’ (heels, sandals, and slippers) [46]. We also observed a trend where females were more likely to report footwear as causing the fall than males (Table 2). Overall, the differences for young adult females observed here lead to the recommendation that gait protocols should be developed to identify sex-related differences not only in older adults, but also in younger adults. Sex-related differences have been examined during steady state gait [47–51], but should also be extended to adaptive gait (e.g. obstacle crossing, stair climbing), gaze behavior during adaptive gait, perturbed gait, and perturbation training to further understand sex-related locomotor differences. In addition, the sex-related differences observed here indicate that fall prevention interventions will likely need to sex-specific. For example, consider the following observations from the current study and previous observations: 1) females reported a higher number of trips (Table 3), females were more likely than males to sustain an injury from a trip [2], and trips are a common cause of falls (Table 2; [19, 21–23]). These observations support further exploration of trips in females.

The high percentage of falls on stairs—13% of all falls, or 22% of non-sports related falls—are especially compelling given that traversing 60 flights of stairs a day accounts for only 1% of an 18-hour day (conservative estimate of 10 s per flight of stairs). Stairs are common locations for falls and fall-related injuries across the lifespan [2, 3, 52]. Half of the texting-related falls occurred on stairs, likely reflecting impaired gait behavior when attention is divided and/or gaze is diverted (e.g. [53–55]). The high percentage of falls on stairs observed here is consistent with the peak in injury rate due to stair-related falls for ages 21–30 years [3]. Furthermore, the trend for higher percentage of falls on stairs for females (15% vs 10%, Table 2) is consistent with higher injuries resulting from stair-related falls in females [3]. These observations demonstrate the need for continued research on stair behavior (e.g. [54, 56, 57]) as well as improved stair safety through environmental modifications (e.g. [58, 59]).

Consistent with research demonstrating that alcohol increases risks for falls (e.g. [60–63]), 11% of falls in the young adults observed here were reported as associated with alcohol/drugs (Table 2). The number of falls associated with alcohol/drugs may be higher than reported, due to the effects of intoxication on recall accuracy (e.g. [64]). Conversely, since college students are more likely to participate in heavy/binge drinking than their non-college attending peers [65–67], the alcohol-related falls reported here may be higher than that observed in the general population of young adults. Despite the clear association between alcohol and falls across the lifespan [5, 60–63], fall questionnaires/diaries do not always quantify the role of alcohol/recreational drugs (e.g. [2, 9, 19, 21–23]). Overall, fall-related research should include alcohol/recreational drugs when quantifying the circumstances of falls across the adult lifespan.

There are several limitations to this study. Self-report fall surveys are affected by recall bias [17, 18, 68, 69], but self-report is currently the only viable approach for examining fall frequency and circumstances in community-dwelling adults. For number of falls, we expect that daily surveys would mitigate the effect of recall bias on the number of falls [69]. However, reporting fall circumstances, such as what caused the fall, may have been affected by recall bias, as has been observed for older adults [68]. A promising future approach includes wearable devices that detect falls and daily activities, as the accuracy of these devices continues to improve (e.g. [70]). In low frequency measures, such as injuries, power may not have been adequate to detect sex-related differences. Similarly, the number of subjects who were taking three or more medications was relatively low (18 out of 325 participants, Fig 5), but we note that the significant increase in number of falls was also observed from 0 to 1 medication, and from 1 and 2 medications. Follow-up research with larger numbers of participants is needed to ensure the observed associations are not spurious. Medication use was examined as number of prescription medications [15, 16], but more information may have been gained by examining categories of medications and dosages. Information on physical activity and medications was assessed once in the initial survey at baseline, but these measures may have changed over the observation period. For example, physical activity is affected by the change in seasons (e.g. [71, 72]). Finally, undergraduate students provide a convenience sample, but are not representative of all young adults aged 18–27 years. Future research should examine more heterogeneous groups.

## Summary

This study observed that frequency of falls was positively associated with physical activity level, number of prescription medications, and being male in young adults. While falls in older adults have been extensively examined, falls in young adults also occur frequently and should not be considered trivial. Since physical activity increased fall-risk in young adults, but is generally protective of falls in older adults, understanding the relationship between falls and physical activity across the lifespan is a critical area for developing interventions to mitigate falls in all adults. It is important to further explore the relationship between falls and number of medications given that it is consistent across the adult lifespan. The results here did not definitively identify why females sustain more fall-related injuries than males (Fig 1), but possibilities for further exploration were identified, including higher likelihood of tripping and impaired ability to walk while talking.

## References

[1] Court-Brown CM, Clement ND, Duckworth AD, Biant LC, McQueen MM. The changing epidemiology of fall-related fractures in adults. Injury. 2017;48: 819–824. 10.1016/j.injury.2017.02.021 28283181

[2] Timsina LR, Willetts JL, Brennan MJ, Marucci-Wellman H, Lombardi DA, Courtney TK, et al. Circumstances of fall-related injuries by age and gender among community-dwelling adults in the United States. PLoS One. 2017;12: e0176561. 10.1371/journal.pone.0176561 28472065 PMC5417511

[3] Blazewick DH, Chounthirath T, Hodges NL, Collins CL, Smith GA. Stair-related injuries treated in United States emergency departments. Am J Emerg Med. 2018;36: 608–614. 10.1016/j.ajem.2017.09.034 28947224

[4] Heijnen MJH, Rietdyk S. Falls in young adults: Perceived causes and environmental factors assessed with a daily online survey. Hum Mov Sci. 2016;46: 86–95. 10.1016/j.humov.2015.12.007 26741254

[5] James MK, Victor MC, Saghir SM, Gentile PA. Characterization of fall patients: Does age matter? J Safety Res. 2018;64: 83–92. 10.1016/j.jsr.2017.12.010 29636172

[6] Verma SK, Willetts JL, Corns HL, Marucci-Wellman HR, Lombardi DA, Courtney TK. Falls and fall-related injuries among community-dwelling adults in the United States. PLoS One. 2016;11: e0150939. 10.1371/journal.pone.0150939 26977599 PMC4792421

[7] CDC. Centers for Disease Control and Prevention. Web-based Injury Statistics Query and Reporting System (WISQARS): nonfatal injury reports.

[8] Stevens JA, Sogolow ED. Gender differences for non-fatal unintentional fall related injuries among older adults. Inj Prev. 2005;11: 115–119. 10.1136/ip.2004.005835 15805442 PMC1730193

[9] Talbot LA, Musiol RJ, Witham EK, Metter EJ. Falls in young, middle-aged and older community dwelling adults: Perceived cause, environmental factors and injury. BMC Public Health. 2005;5: 86. 10.1186/1471-2458-5-86 16109159 PMC1208908

[10] Westerterp KR, Meijer EP. Physical activity and parameters of aging: A physiological perspective. Journals Gerontol—Ser A Biol Sci Med Sci. 2001;56: 7–12. 10.1093/gerona/56.suppl_2.7 11730240

[11] Krems C, Luhrmann PM, Neuhauser-Berthold M. Physical activity in young and elderly subjects. Journal of sports medicine and physical fitness. J Sports Med Phys Fitness. 2004;44: 71. 15181393

[12] Peeters GMEE, Verweij LM, Van Schoor NM, Pijnappels M, Pluijm SMF, Visser M, et al. Which types of activities are associated with risk of recurrent falling in older persons? Journals Gerontol—Ser A Biol Sci Med Sci. 2010;65 A: 743–750. 10.1093/gerona/glq013 20159779

[13] Piercy KL, Troiano RP, Ballard RM, Carlson SA, Fulton JE, Galuska DA, et al. The physical activity guidelines for Americans. JAMA—J Am Med Assoc. 2018;320: 2020–2028. 10.1001/jama.2018.14854 30418471 PMC9582631

[14] De Jong MR, Van Der Elst M, Hartholt KA. Drug-related falls in older patients: Implicated drugs, consequences, and possible prevention strategies. Ther Adv Drug Saf. 2013;4: 147–154. 10.1177/2042098613486829 25114778 PMC4125318

[15] Laflamme L, Monárrez-Espino J, Johnell K, Elling B, Möller J. Type, number or both? A population-based matched case-control study on the risk of fall injuries among older people and number of medications beyond fall-inducing drugs. PLoS One. 2015;10: 1–12. 10.1371/journal.pone.0123390 25815483 PMC4376700

[16] Kool B, Ameratunga S, Robinson E. Association between prescription medications and falls at home among young and middle-aged adults. Inj Prev. 2012;18: 200–203. 10.1136/injuryprev-2011-040202 22248914

[17] Cummings SR, Nevitt MC, Kidd S. Forgetting Falls. J Am Geriatr Soc. 1988;36: 613–616. 10.1111/j.1532-5415.1988.tb06155.x 3385114

[18] Ganz DA, Higashi T, Rubenstein LZ. Monitoring falls in cohort studies of community-dwelling older people: Effect of the recall interval. J Am Geriatr Soc. 2005;53: 2190–2194. 10.1111/j.1532-5415.2005.00509.x 16398908

[19] Hill K, Schwarz J, Flicker L, Carroll S. Falls among healthy, community-dwelling, older women: a prospective study of frequency, circumstances, consequences and prediction accuracy. Aust N Z J Public Health. 1999;23: 41–48. 10.1111/j.1467-842x.1999.tb01203.x 10083688

[20] Peel N. Validating recall of falls by older people. Accid Anal Prev. 2000;32: 371–372. 10.1016/s0001-4575(99)00066-4 10776852

[21] Berg WP, Alessio HM, Mills EM, Tong C. Circumstances and consequences of falls in independent community-dwelling older adults. Age Ageing. 1997;26: 261–268. 10.1093/ageing/26.4.261 9271288

[22] Blake AJ, Morgan K, Bendall MJ, Dallosso H, Ebrahim SBJ, Arie THD, et al. Falls by elderly people at home: prevalence and associated factors. Age Ageing. 1988;17: 365–372. 10.1093/ageing/17.6.365 3266440

[23] Duckham RL, Procter-Gray E, Hannan MT, Leveille SG, Lipsitz LA, Li W. Sex differences in circumstances and consequences of outdoor and indoor falls in older adults in the MOBILIZE Boston cohort study. BMC Geriatr. 2013;13. 10.1186/1471-2318-13-133 24313971 PMC3907046

[24] Persell SD, Osborn CY, Richard R, Skripkauskas S, Wolf MS. Limited health literacy is a barrier to medication reconciliation in ambulatory care. J Gen Intern Med. 2007;22: 1523–1526. 10.1007/s11606-007-0334-x 17786521 PMC2219798

[25] Godin G, Shephard RJ, others. A simple method to assess exercise behavior in the community. Can J Appl Sport Sci. 1985;10: 141–146. 4053261

[26] Mâsse LC, de Niet JE. Sources of Validity Evidence Needed with Self-Report Measures of Physical Activity. J Phys Act Heal. 2016;9: S44–S55. 10.1123/jpah.9.s1.s44 22287447

[27] Weerdesteyn V, Rijken H, Geurts ACH, Smits-Engelsman BCM, Mulder T, Duysens J. A five-week exercise program can reduce falls and improve obstacle avoidance in the elderly. Gerontology. 2006;52: 131–141. 10.1159/000091822 16645293

[28] Verma SK, Chang WR, Courtney TK, Lombardi DA, Huang YH, Brennan MJ, et al. Workers’ experience of slipping in U.S. limited-service restaurants. J Occup Environ Hyg. 2010;7: 491–500. 10.1080/15459624.2010.486693 20552500

[29] Muir BC, Bodratti LA, Morris CE, Haddad JM, van Emmerik REA, Rietdyk S. Gait characteristics during inadvertent obstacle contacts in young, middle-aged and older adults. Gait Posture. 2020;77: 100–104. 10.1016/j.gaitpost.2020.01.020 32006717

[30] Allin LJ, Brolinson PG, Beach BM, Kim S, Nussbaum MA, Roberto KA, et al. Perturbation-based balance training targeting both slip- And trip-induced falls among older adults: A randomized controlled trial. BMC Geriatr. 2020;20: 1–13. 10.1186/s12877-020-01605-9 32532221 PMC7291462

[31] Lee J. The association between physical activity and risk of falling in older adults: A systematic review and meta-analysis of prospective cohort studies. Geriatr Nurs. 2020;41. 10.1016/j.gerinurse.2020.05.005 32507370

[32] Freedman VA, Hodgson N, Lynn J, Spillman BC, Waidmann T, Wilkinson AM, et al. Promoting declines in the prevalence of late-life disability: Comparisons of three potentially high-impact interventions. Milbank Q. 2006;84: 493–520. 10.1111/j.1468-0009.2006.00456.x 16953808 PMC2690252

[33] Pereira CLN, Baptista F, Infante P. Role of physical activity in the occurrence of falls and fall-related injuries in community-dwelling adults over 50 years old. Disabil Rehabil. 2014;36: 117–124. 10.3109/09638288.2013.782355 23594055

[34] Gebel K, Ding D, Chey T, Stamatakis E, Brown WJ, Bauman AE. Effect of moderate to vigorous physical activity on all-cause mortality in middle-aged and older Australians. JAMA Intern Med. 2015;175: 970–977. 10.1001/jamainternmed.2015.0541 25844882

[35] Morin L, Larrañaga AC, Welmer AK, Rizzuto D, Wastesson JW, Johnell K. Polypharmacy and injurious falls in older adults: A nationwide nested case-control study. Clin Epidemiol. 2019;11: 483–493. 10.2147/CLEP.S201614 31296999 PMC6598933

[36] Heijnen MJH, Muir BC, Rietdyk S. Factors leading to obstacle contact during adaptive locomotion. Exp Brain Res. 2012;223: 219–231. 10.1007/s00221-012-3253-y 22972450

[37] Chou LS, Draganich LF. Placing the trailing foot closer to an obstacle reduces flexion of the hip, knee, and ankle to increase the risk of tripping. J Biomech. 1998;31: 685–691. 10.1016/s0021-9290(98)00081-5 9796668

[38] Lundeberg MA, Fox PW, Punćochaŕ J. Highly Confident but Wrong: Gender Differences and Similarities in Confidence Judgments. J Educ Psychol. 1994;86: 114–121. 10.1037/0022-0663.86.1.114

[39] Lundin-olsson L, Nyberg L, Gustafson Y. “Stops walking when talking” as a predictor of falls in elderly people. Lancet. 1997;349: 617. 10.1016/S0140-6736(97)24009-2 9057736

[40] Raffegeau TE, Haddad JM, Huber JE, Rietdyk S. Walking while talking: Young adults flexibly allocate resources between speech and gait. Gait Posture. 2018;64: 59–62. 10.1016/j.gaitpost.2018.05.029 29859413

[41] Beliefs Yagil D., motives and situational factors related to pedestrians’ self-reported behavior at signal-controlled crossings. Transp Res Part F Traffic Psychol Behav. 2000;3: 1–13. 10.1016/S1369-8478(00)00004-8

[42] Tom A, Granié MA. Gender differences in pedestrian rule compliance and visual search at signalized and unsignalized crossroads. Accid Anal Prev. 2011;43: 1794–1801. 10.1016/j.aap.2011.04.012 21658507

[43] Neder JA, Nery LE, Silva AC, Andreoni S, Whipp BJ. Maximal aerobic power and leg muscle mass and strength related to age in non-athletic males and females. Eur J Appl Physiol Occup Physiol. 1999;79: 522–530. 10.1007/s004210050547 10344462

[44] Der G, Deary IJ. Age and sex differences in reaction time in adulthood: Results from the United Kingdom health and lifestyle survey. Psychol Aging. 2006;21: 62–73. 10.1037/0882-7974.21.1.62 16594792

[45] Nieves JW, Formica C, Ruffing J, Zion M, Garrett P, Lindsay R, et al. Males have larger skeletal size and bone mass than females, despite comparable body size. J Bone Miner Res. 2005;20: 529–535. 10.1359/JBMR.041005 15746999

[46] Dufour AB, Broe KE, Nguyen USDT, Gagnon DR, Hillstrom HJ, Walker AH, et al. Foot pain: Is current or past shoewear a factor? Arthritis Care Res. 2009;61: 1352–1358. 10.1002/art.24733 19790125 PMC2761974

[47] Cho SH, Park JM, Kwon OY. Gender differences in three dimensional gait analysis data from 98 healthy Korean adults. Clin Biomech. 2004;19: 145–152. 10.1016/j.clinbiomech.2003.10.003 14967577

[48] Kerrigan DC, Todd MK, Della Croce U. Gender differences in joint biomechanics during walking: Normative study in young adults. American Journal of Physical Medicine and Rehabilitation. 1998. pp. 2–7. 10.1097/00002060-199801000-00002 9482373

[49] Ko S-U, Tolea MI, Hausdorff JM, Ferrucci L. Sex-specific differences in gait patterns of healthy older adults. J Biomech. 2011;44: 1974–1979. 10.1016/j.jbiomech.2011.05.005 21601861 PMC3124580

[50] Nissan M, Whittle MW. Initiation of gait in normal subjects: a preliminary study. J Biomed Eng. 1990;12: 165–171. 10.1016/0141-5425(90)90139-e 2319768

[51] Winter DA. Biomechanics and motor control of human gait: normal, elderly and pathological. 1991.

[52] Jacobs J V. A review of stairway falls and stair negotiation: Lessons learned and future needs to reduce injury. Gait Posture. 2016;49: 159–167. 10.1016/j.gaitpost.2016.06.030 27427833

[53] Miyasike-daSilva V, McIlroy WE. Gaze shifts during dual-tasking stair descent. Exp Brain Res. 2016;234: 3233–3243. 10.1007/s00221-016-4721-6 27401474 PMC5071366

[54] Telonio A, Blanchet S, Maganaris CN, Baltzopoulos V, Villeneuve S, McFadyen BJ. The division of visual attention affects the transition point from level walking to stair descent in healthy, active older adults. Exp Gerontol. 2014;50: 26–33. 10.1016/j.exger.2013.11.007 24291246

[55] Cho H, Romine NL, Barbieri FA, Rietdyk S. Gaze diversion affects cognitive and motor performance in young adults when stepping over obstacles. Gait Posture. 2019;73: 273–278. 10.1016/j.gaitpost.2019.07.380 31394370

[56] Den Otter AR, Hoogwerf M, Van Der Woude LH. The role of tread fixations in the visual control of stair walking. Gait Posture. 2011;34: 169–173. 10.1016/j.gaitpost.2011.04.004 21550247

[57] Zietz D, Hollands M. Gaze behavior of young and older adults during stair walking. J Mot Behav. 2009;41: 357–365. 10.3200/JMBR.41.4.357-366 19508962

[58] Foster RJ, Hotchkiss J, Buckley JG, Elliott DB. Safety on stairs: Influence of a tread edge highlighter and its position. Exp Gerontol. 2014;55: 152–158. 10.1016/j.exger.2014.04.009 24768822

[59] Thomas NM, Skervin T, Foster RJ, O’Brien TD, Carpenter MG, Maganaris CN, et al. Optimal lighting levels for stair safety: Influence of lightbulb type and brightness on confidence, dynamic balance and stepping characteristics. Exp Gerontol. 2020;132: 110839. 10.1016/j.exger.2020.110839 31958491

[60] Hingson R, Howland J. Alcohol as a risk factor for injury or death resulting from accidental falls: a review of the literature. J Stud Alcohol. 1987;48: 212–219. 10.15288/jsa.1987.48.212 3309468

[61] McErlean DR, Hughes JA. Who falls in an adult emergency department and why—A retrospective review. Australas Emerg Nurs J. 2017;20: 12–16. 10.1016/j.aenj.2016.11.001 28034556

[62] Kool B, Ameratunga S, Jackson R. The role of alcohol in unintentional falls among young and middle-aged adults: A systematic review of epidemiological studies. Inj Prev. 2009;15: 341–347. 10.1136/ip.2008.021303 19805604

[63] Chen CM, Yoon YH. Usual Alcohol Consumption and Risks for Nonfatal Fall Injuries in the United States: Results From the 2004–2013 National Health Interview Survey. Subst Use Misuse. 2017;52: 1120–1132. 10.1080/10826084.2017.1293101 28524713 PMC6080198

[64] Schreiber Compo N, Carol RN, Evans JR, Pimentel P, Holness H, Nichols-Lopez K, et al. Witness memory and alcohol: The effects of state-dependent recall. Law Hum Behav. 2017;41: 202–215. 10.1037/lhb0000224 27786509

[65] Hingson RW, Heeren T, Zakocs RC, Kopstein A, Wechsler H. Magnitude of alcohol-related mortality and morbidity among U.S. College students ages 18–24. J Stud Alcohol. 2002;63: 136–144. 10.15288/jsa.2002.63.136 12033690

[66] Johnston LD, O’Malley PM, Bachman JG. Monitoring the Future national survey results on drug use, 1975–2002. Volume II: College students and adults ages 19–40. Bethesda, MD: National Institute on Drug Abuse; 2003.

[67] Slutske WS, Hunt-Carter EE, Nabors-Oberg RE, Sher KJ, Bucholz KK, Madden PAF, et al. Do college students drink more than their non-college-attending peers? Evidence from a population-based longitudinal female twin study. J Abnorm Psychol. 2004;113: 530–540. 10.1037/0021-843X.113.4.530 15535786

[68] Yang Y, Feldman F, Leung PM, Scott V, Robinovitch SN. Agreement between video footage and fall incident reports on the circumstances of falls in long-term care. J Am Med Dir Assoc. 2015;16: 388–394. 10.1016/j.jamda.2014.12.003 25669670

[69] Hannan MT, Gagnon MM, Aneja J, Jones RN, Cupples LA, Lipsitz LA, et al. Optimizing the tracking of falls in studies of older participants: Comparison of quarterly telephone recall with monthly falls calendars in the MOBILIZE Boston study. Am J Epidemiol. 2010;171: 1031–1036. 10.1093/aje/kwq024 20360242 PMC2877474

[70] Gjoreski M, Gjoreski H, Luštrek M, Gams M. How accurately can your wrist device recognize daily activities and detect falls? Sensors. 2016;16. 10.3390/s16060800 27258282 PMC4934226

[71] Yoshimura E, Tajiri E, Hatamoto Y, Tanaka S. Changes in Season Affect Body Weight, Physical Activity, Food Intake, and Sleep in Female College Students: A Preliminary Study. Int J Environ Res Public Health. 2020;17: 8713. 10.3390/ijerph17238713 33255205 PMC7727682

[72] Westerterp KR. Seasonal variation in body mass, body composition and activity-induced energy expenditure: a long-term study. Eur J Clin Nutr. 2020;74: 135–140. 10.1038/s41430-019-0408-y 30787469

